# Design of Soft, Stretchable Bladder-Integrated Scaffolds for Advanced Bioelectronic Implants

**DOI:** 10.1002/admt.202500552

**Published:** 2025-07-02

**Authors:** Yifan Wang, Ali Garmroudi, Chang Liu, Philippe Zimmern, Zhengwei Li

**Affiliations:** Department of Biomedical Engineering, University of Houston, Houston, TX 77204, USA; Department of Biomedical Engineering, University of Houston, Houston, TX 77204, USA; Department of Biomedical Engineering, University of Houston, Houston, TX 77204, USA; Department of Urology, The University of Texas Southwestern, Dallas, TX 75390, USA; Department of Biomedical Engineering, University of Houston, Houston, TX 77204, USA; Department of Biomedical Sciences, The Tilman J. Fertitta Family College of Medicine, University of Houston, Houston, TX 77021, USA; Institute of Muscle Biology and Cachexia, University of Houston, Houston, TX 77204, USA

**Keywords:** advanced urotechnology, bioelectronic implants, engineering design, flexible electronics, neurogenic bladder, soft materials

## Abstract

Neurogenic bladder is a challenging condition caused by neurological disorders, such as spinal cord injury (SCI), leading to impaired bladder function. Bladder-integrated implants are critically needed for restoring function, managing incontinence, or continuous bladder health monitoring. However, developing such neurotechnology remains challenging due to the bladder’s large and dynamic volume changes (≈300%), which often cause mechanical incompatibility, tissue irritation, and limited long-term efficacy. In this work, it is designed and evaluate highly stretchable, biocompatible, and bladder-integrated implantable scaffolds that seamlessly conform to bladder expansion and contraction with negligible physiological impact using a biomimetic in vitro bladder model. This findings show that cross-shaped scaffolds provide superior stretch-ability with negligible effect on bladder compliance. Compared to the control (no implants), these scaffolds exhibit minimum effect on bladder deformation even under 300% volume expansion. Long-term mechanical tests confirm their positional stability, while cytocompatibility studies show high biocompatibility (>99.5% cell viability), highlighting their potential usage for chronic implantation in urological applications. This work provides important insights into the development of advanced bladder-integrated, highly stretchable bioelectronic implants for real-time monitoring and neuromodulation of bladder dysfunction. The proposed novel engineering design and innovative approach lay a solid foundation for next-generation bladder rehabilitation technologies.

## 1. Introduction

Neurogenic lower urinary tract dysfunction (NLUTD) is a common condition resulting from neurological disorders such as spinal cord injury (SCI), multiple sclerosis, Parkinson’s disease, and other neu-rodegenerative diseases.^[1,2]^ NLUTD impairs bladder function, leading to severe complications such as bladder areflexia, detrusor overactivity, and increased risk of recurrent infections and kidney damage, which affects ≈70%–84% of individuals with SCI.^[3]^ Current management strategies primarily include intermittent catheterization,^[4,5]^ pharmacological treatments such as antimuscarinics^[6]^ and beta-3 agonists,^[7]^ and surgical interventions like sphincterotomy^[8,9]^ and urethral stents.^[10]^ However, these methods often result in limited efficacy, significant side effects, and poor long-term outcomes, which highlight the need for improved therapeutic solutions.^[10–12]^

**Figure 1.**
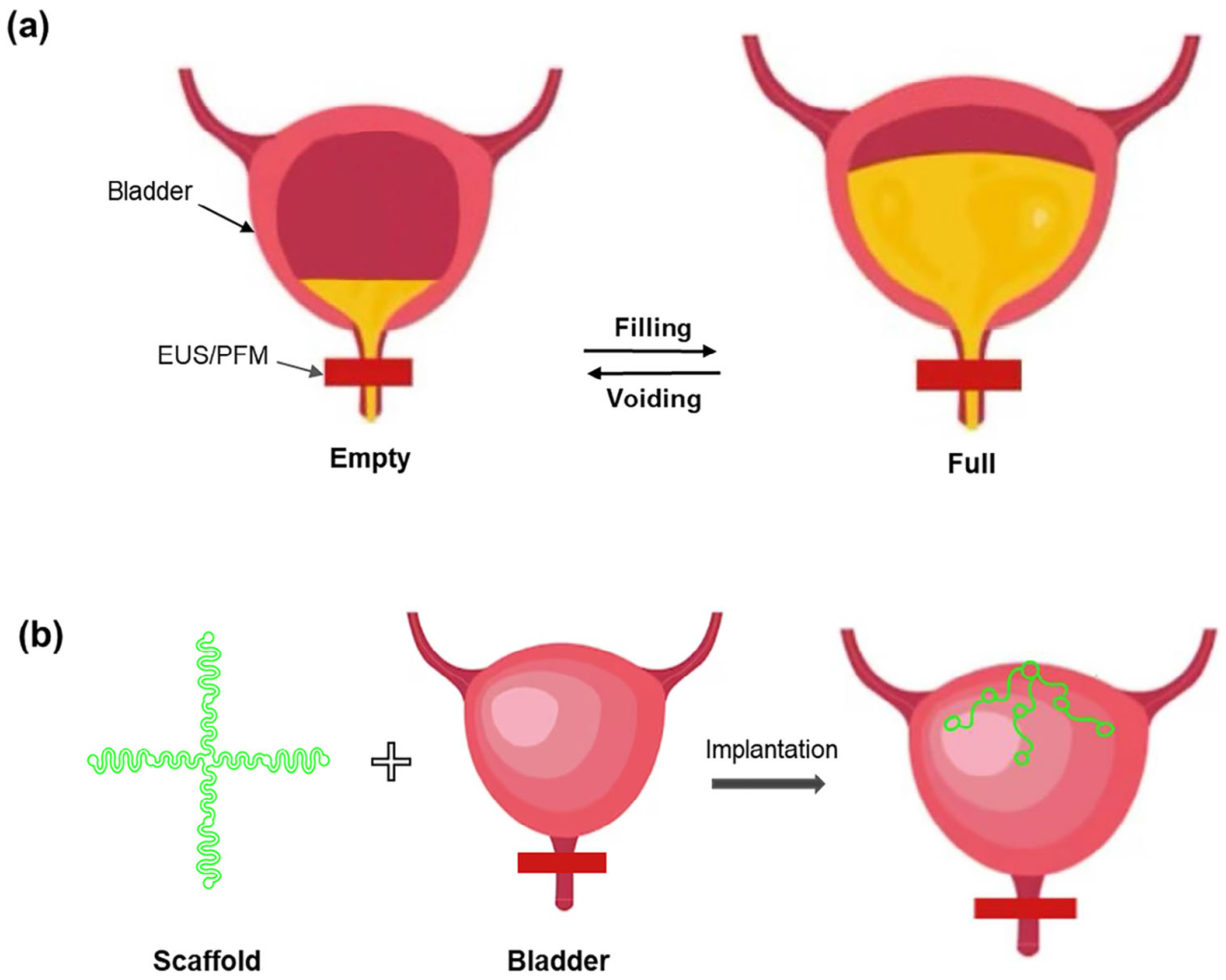
Design and integration of soft, highly stretchable bladder implants. a) Schematic of the dynamic urinary filling and voiding process. b) Schematic representation of the implantation process, where soft, stretchable scaffolds are integrated onto the outer wall of the bladder to accommodate dynamic expansion and contraction.

The bladder is a highly dynamic organ that undergoes significant volume and shape changes during the filling and voiding cycles^[13,14]^ (Figure 1a). These unique biomechanical properties present substantial challenges in developing effective technologies for monitoring and neuromodulation therapy. Existing approaches, such as catheter-based pressure sensors^[15]^ and wearable ultrasound devices,^[16,17]^ offer limited real-time data and are either invasive or impractical for continuous monitoring.^[18]^ Electrical neuromodulation techniques, including sacral and tibial nerve stimulation,^[19–21]^ show promise in restoring bladder control but lack precise bladder-targeted stimulation, leading to inconsistent therapeutic outcomes. Additionally, the rigid nature of conventional electronics makes them incompatible with the bladder’s extreme mechanical deformations, often resulting in mechanical failure and poor long-term performance.

Bladder-integrated implants hold great potential for providing real-time health monitoring and direct neuromodulation. However, their design is extremely challenging due to the bladder’s biomechanical complexity. The bladder undergoes extreme volumetric changes (≈300%) and deformation during normal physiological function, subjecting any implanted device to substantial mechanical stress. Traditional rigid electronics fail to accommodate these changes, leading to failure, irritation, or interference with normal bladder function. To overcome these challenges, advanced engineering solutions are required to develop bladder-integrated implants that can function reliably without affecting bladder physiology.

Recent advancements in the field of soft and stretchable electronics^[22–25]^ have yielded numerous implantable systems that interface intimately with internal organs.^[26–28]^ These advanced bioelectronic systems have been designed to integrate with the heart,^[29,30]^ brain,^[31]^ and other organs,^[22]^ exhibiting superior mechanical and electrical properties, such as high stretchability, mechanical robustness, and long-term durability. Notably, recent studies have introduced bladder-integrated bioelectronics, including an expandable and implantable bioelectronic system capable of real-time bladder monitoring and optoelectronic stimulation^[32]^ along with a wireless, fully implantable electronic system for bidirectional electrical neuromodulation of the bladder.^[26]^ Although these studies represent significant advancements, they do not comprehensively investigate the influence of bioelectronic implants on bladder function (such as bladder tissue deformation, volume change, etc.), particularly in response to its dynamic expansion and contraction cycles.

The bladder’s unique biomechanical properties, including its large and dynamic volume changes (≈300%), present fundamental challenges for implant design. Unlike other soft tissues, the bladder undergoes repeated and extreme mechanical deformations, which can compromise the long-term stability and functionality of implanted devices. A successful bladder-integrated system must exhibit high mechanical compliance, maintain stable adhesion under constant volumetric shifts, and ensure minimal disruption to normal bladder physiology. These challenges highlight the need for advanced engineering solutions that optimize both material properties and structural designs for seamless bladder integration. Recent innovations in stretchable bioelectronics have demonstrated that structural design along with material selection are critical for successful integration with soft and deformable organs.^[33,34]^ Design strategies such as serpentine interconnects,^[35]^ porous and wavy layouts^[36,37]^ and origami/kirigami-inspired architectures^[38,39]^ have enabled high levels of mechanical compliance and conformability. These geometries help reduce strain on functional components while maintaining intimate tissue contact—properties essential for dynamic environments like the bladder. Among these, open-mesh designs have emerged as a promising class for covering large organ surfaces while minimizing mechanical constraint. Our proposed cross-shaped scaffold adopts this open-mesh design principle and is specifically engineered for the bladder’s extreme volumetric changes (Figure 1b). To contextualize this approach, we summarize representative prior studies using advanced materials and structural configurations, including mesh, coil, and kirigami structures, along with their reported stretchability performance in Table S1 (Supporting Information). These insights highlight the relevance of structural mechanics in bioelectronics and support the novelty of our design in addressing bladder-specific challenges.

In this work, we propose optimized engineering designs for highly stretchable and implantable scaffolds that seamlessly integrate with the bladder. Using soft, flexible materials, we engineer the scaffolds to conform to the bladder’s shape while preserving its natural expansion and contraction cycles. A biomimetic in vitro bladder model (i.e., water balloon) is employed to systematically examine implant flexibility, contact area (coverage), and long-term mechanical stability. Additionally, we conduct cell viability tests and demonstrate a high biocompatibility of the fabricated soft scaffolds, which will serve as soft scaffolds for advanced bladder-implantable bioelectronics. Through optimizing material properties, scaffold geometry, and integration strategies, we ensure minimal physiological interference and robust adhesion under dynamic volumetric changes. This work advances bladder-integrated bioelectronics by providing a scalable platform for monitoring and neuromodulation applications, and providing important insights for future preclinical and clinical applications in other urological applications.

## 2. Results and Discussion

### 2.1. Design and Integration of Bladder Scaffolds

The mechanical flexibility and seamless integration of bladder implants are essential for their long-term functionality. These implants must be highly stretchable to accommodate bladder expansion, and simultaneously exert negligible impact on bladder mechanics, including volume changes and bladder deformation, to preserve normal function. Innovative mechanical designs, particularly open-mesh serpentine structures, have facilitated the integration of electronic devices with soft tissues in a conformal manner.^[26,30]^ However, designing bladder implants presents unique challenges due to the extreme and dynamic volumetric changes (≈300%) of the urinary bladder. To systematically evaluate the mechanical adaptability of these implants, their stretchability and integration effects are studied using a biomimetic in vitro bladder model, represented by an expandable latex water balloon. To investigate integration effects, two mounting strategies are employed on the biomimetic bladder model. In the partial integration approach, only the “island” sites are bonded to the surface of a latex water balloon using a thin layer of uncured silicone elastomer (Ecoflex or Polydimethylsiloxane (PDMS)), while the serpentine interconnects remain free to move. In the full integration method, the entire scaffold including interconnects are adhered to the balloon surface using the same uncured silicone, followed by curing at room temperature. These mounting strategies are designed to mimic potential surgical approaches and assess their influence on bladder mechanics. They also serve as a practical reference for future in vivo applications where bioadhesives may offer advantages over sutures in minimizing tissue trauma.

**Figure 2.**
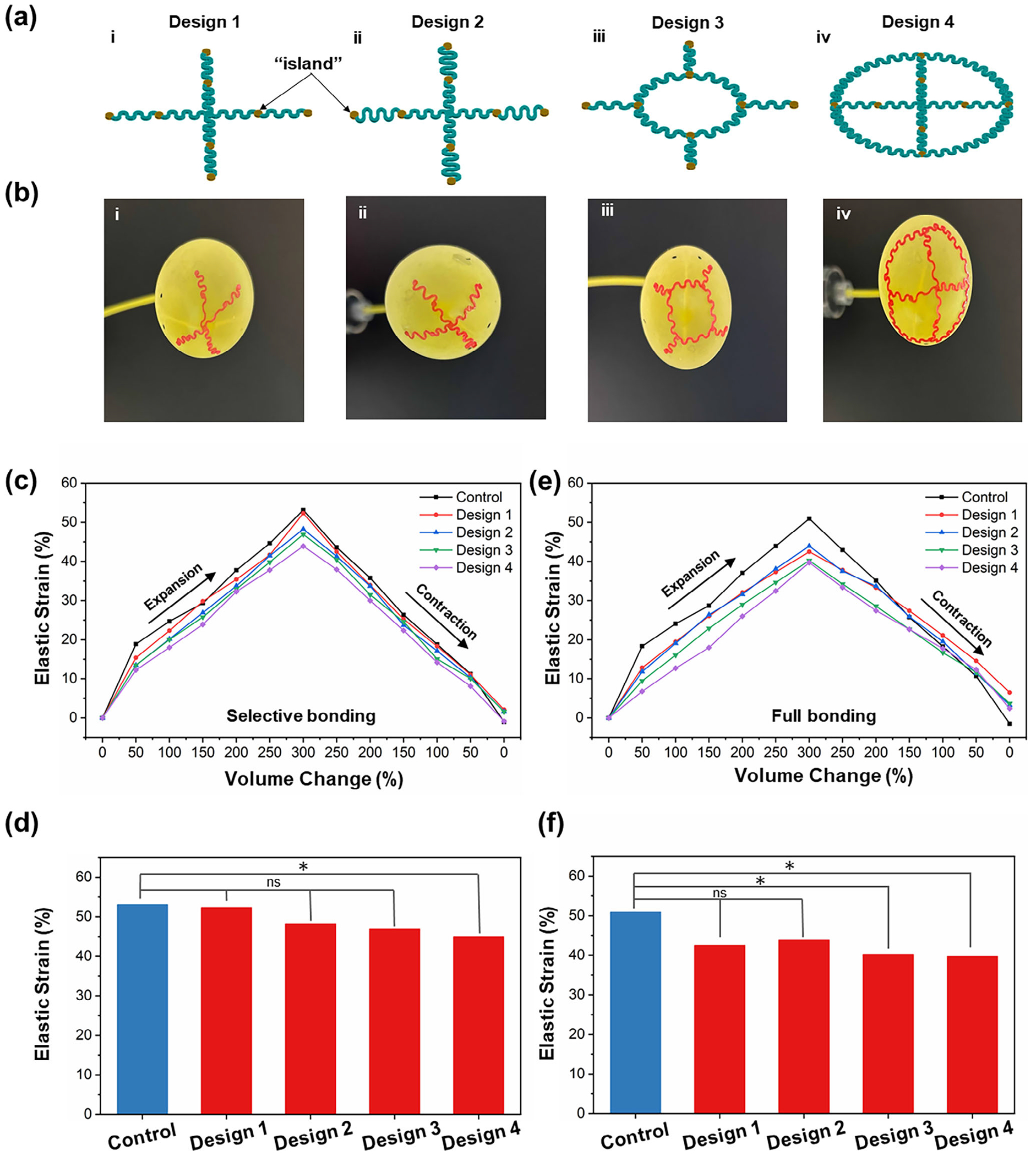
Design and integration of bladder scaffolds. a) Schematic of four scaffold designs for bladder integration: i, ii) cross-shaped scaffolds with perpendicular serpentine interconnects and iii, iv) web-like scaffolds with a central or circumferential structure. The “island” sites serve as attachment points for microelectrodes and biosensors. b) Scaffolds mounted on a biomimetic bladder model (latex water balloon) to evaluate mechanical effects under dynamic expansion. c) Loading history of bladder elastic strain for different scaffold designs (mounted only at island sites) and the control (no implant) during the expansion and contraction process, mimicking the bladder filling and voiding process, and d) Comparison of elastic strain differences in bladder expansion between scaffolds and the control under 300% volume change. e) Elastic strain variations during the filling-voiding cycle with fully integrated scaffolds (Note: Residual strain observed in some groups may result from implant-induced mechanical constraints and minor image-based measurement variations.). f) Comparative analysis of mechanical constraints across different scaffold configurations when the entire scaffold is mounted.

Four different engineering scaffold designs are proposed and examined to assess their mechanical effects on bladder expansion, including two cross-shaped (Figure 2a–i,ii) and two web-like (Figure 2a–iii,iv) configurations. The designs consist of eight “island” sites (diameter: 1.5 mm) that serve as potential regions for microelectrodes and biosensors, interconnected by serpentine interconnects (inner diameter 0.8mm and outer diameter of 2 mm) to provide large stretchability and conformal integration. The cross-shaped designs (Figure 2a–i,ii) incorporate perpendicular serpentine interconnects, allowing expansion in the radial direction. The design in Figure 2a–ii features a larger aspect ratio of wavelength to amplitude in the serpentine interconnects in between the “island” sites compared to Figure 2a–i. In contrast, the web-like scaffolds (Figure 2a–iii,iv) have a central circular structure with radiating wavy interconnects (Figure 2a–iii) or a peripheral circular structure enclosing two perpendicular wavy lines (Figure 2a–iv), both of which offer structural reinforcement.

To investigate the impact of scaffold design on bladder expansion, we mount the implants partially (only at the island sites) or fully on the biomimetic bladder (Figure 2b). Two reference points are marked at the center of the balloon as indicators for quantitatively analyzing the deformation of the “bladder” during expansion. The deformation behavior is assessed under 300% volume inflation and contraction, mimicking the filling and voiding cycle of the urinary bladder. When only the islands are attached, leaving the serpentine interconnects free, the loading history of bladder deformation during the whole expansion and contraction process is obtained in Figure 2c. Cross-like designs (Figure 2a–i,ii) exhibit minimal mechanical constraint, with no statistically significant difference compared to the control (*p >* 0.05). The mechanical constraints for Designs 1, 2, and 3 are 1.49%, 9.14%, and 11.73%, respectively. In this work, mechanical constraint is defined as the difference in bladder elastic strain induced by the implants compared to the control (no implant) at maximum expansion (300% volume change). However, the web-like scaffold (Design 4, Figure 2a–iv), which has a circumferential surrounding structure, imposes a substantially higher mechanical constraint (17.39%), as indicated by the loading history curves in Figure 2c and the strain quantification in Figure 2d.

To further evaluate the effects of full implant integration, we mount the entire scaffold (including serpentine interconnects) onto the water balloon (Figure 2b). The loading curves of bladder elastic strain (Figure 2e) show a greater deviation from the control when the implants are fully bonded, particularly for web-like structures. The mechanical constraints for the four designs are measured as 16.56%, 13.7%, 20.97%, and 22.02%, respectively (Figure 2f). An extreme case is also tested, in which half of the bladder surface is covered with strip scaffolds (Figure S1, Supporting Information), resulting in a significant increase in mechanical constraints (49.12%). It is noted that in certain scaffold designs, particularly those involving full integration or circumferential coverage, non-zero residual strain is observed after unloading. This effect is attributed primarily to mechanical constraints introduced by the implant, which can limit the natural recoil of the bladder during voiding. Additionally, minor contributions from measurement-related factors, such as image-based elastic strain quantification and volumetric control variability during cyclic testing, may also account for small deviations. These findings are consistent with our overall conclusion that scaffold geometry and integration strategy critically influence mechanical compatibility with the bladder.

From a mechanics perspective, the bladder undergoes uniform expansion in both longitudinal and circumferential directions during filling. The web-like designs (Figure 2a–iii,iv), which include interconnects in both longitudinal and circumferential directions, impose higher mechanical constraints on the bladder’s natural expansion. In contrast, the cross-shaped designs (Figure 2a–i,ii) align their serpentine interconnects along the radial direction, allowing the circumferential direction to remain unconstrained. The serpentine interconnects provide bending “room”, which further enhances the stretchability and minimizes the mechanical restriction. Moreover, partial integration (attaching only the island sites) allows the serpentine interconnects to move freely, which significantly reduces mechanical constraints compared to full implantation. The cross-shaped perpendicular interconnects, when partially integrated, enable nearly negligible mechanical constraints, even under large volume changes, which makes them ideal for in vivo applications.

### 2.2. Impact of Scaffold Size on Bladder Expansion

**Figure 3.**
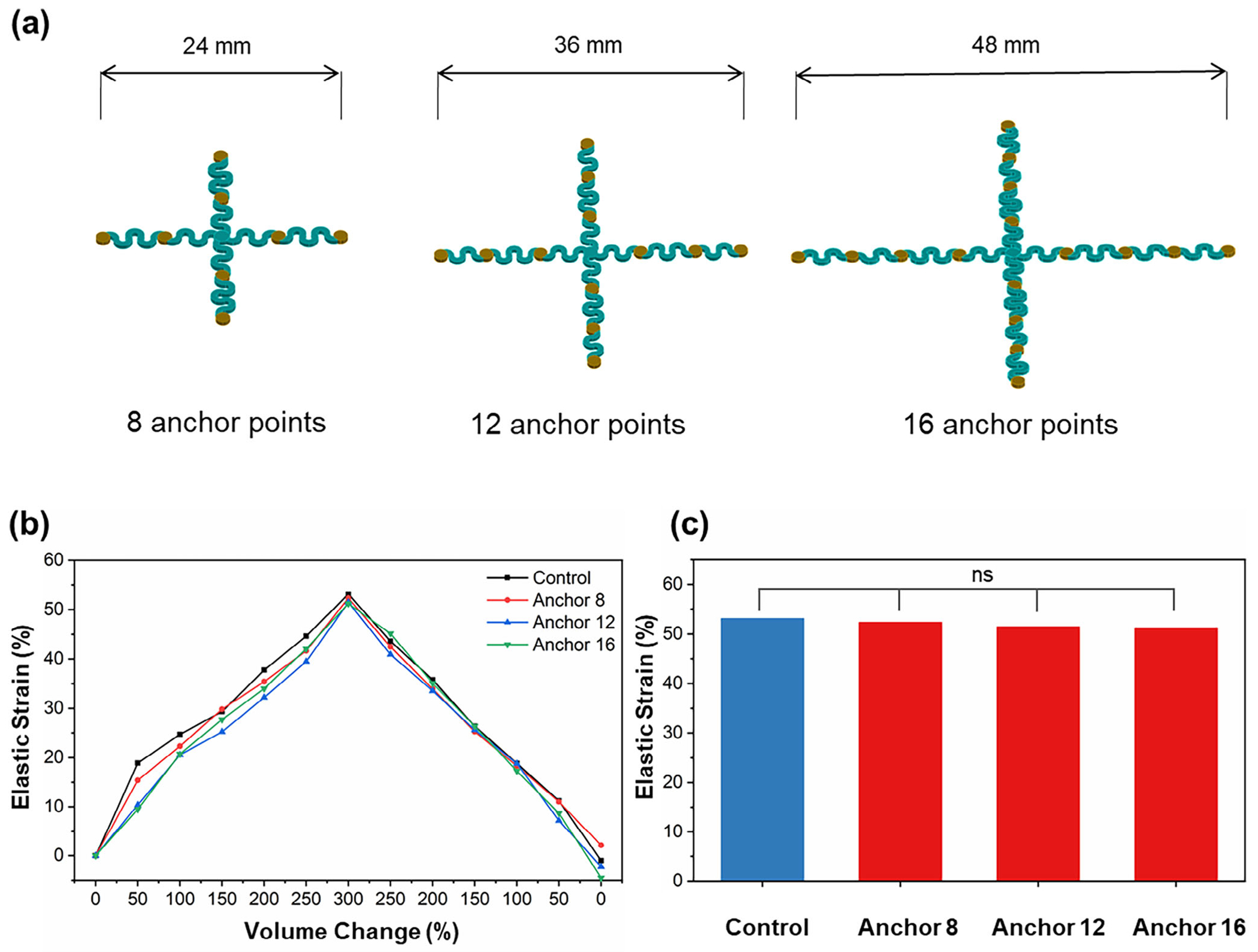
Impact of scaffold size on bladder expansion. a) Schematic of cross-shaped scaffolds with different sizes, featuring 8 islands (24 mm), 12 islands (36 mm), and 16 islands (48 mm) as anchor points. b) Loading history of bladder elastic strain for different scaffold sizes and the control (no implant) during the expansion and contraction process. c) Comparison of elastic strain at 300% volume expansion for different scaffold sizes.

The coverage size of bladder implants is a critical design parameter for the development of next-generation bladder implantable systems. For example, a higher density of microelectrode arrays (MEAs) enables more uniform electrical stimulation on the bladder for effective voiding control, while a higher density of bio-physical sensors enhances bladder mapping and diagnosis of neurogenic bladder dysfunctions. In this section, we evaluate how different sizes of cross-shaped implants affect bladder elastic strain during the filling and voiding process. To systematically investigate the impact of implant size, we select the crossshaped scaffold design and test three variations with different numbers of islands (anchor points): 8 islands (24 mm), 12 islands (36 mm), and 16 islands (48 mm) (Figure 3a). It is remarkable that the results show that all four groups, including the control (no implant), exhibit nearly identical elastic strain-volume change curves (Figure 3b), suggesting that size variations of the scaffold have no significant impact on bladder deformation. Further quantitative analysis at 300% volume expansion confirms that there is no statistically significant difference between scaffold sizes (Figure 3c). The mechanical constraints introduced by the 8-island, 12-island, and 16-island designs were 1.50%, 3.19%, and 3.60%, respectively, compared to the control. These findings suggest that variations in scaffold size and the number of islands have a minimal effect on bladder deformation and overall performance.

As discussed in Section 2.1, the cross-shaped scaffolds bond selectively with bridge sites along the longitudinal direction, leaving the circumferential direction unconstrained. Such selective attachment allows for high stretchability with negligible impact on bladder expansion mechanics. Additionally, the softness of the scaffold material plays a crucial role in stretchability and mechanical performance. Although both Ecoflex and Sylgard 184 PDMS are silicone-based elastomers derived from poly(dimethyl siloxane), they differ significantly in formulation and mechanical properties. As shown in Figure S2a (Supporting Information), Sylgard 184 PDMS exhibits a higher Young’s modulus (≈1.3–2.9 MPa), indicating greater stiffness, whereas Ecoflex demonstrates a significantly lower modulus (≈40–83 kPa), more closely matching the compliance of native bladder tissue. These material distinctions influence the overall stretchability of the implant and the mechanical constraints imposed during bladder expansion. Using the same cross-like design, we examined how material stiffness influences bladder elastic strain during filling and voiding. The results show that softer implants exhibit closer loading curves of bladder elastic deformation to the control, reinforcing the importance of material selection in implant design (Figure S2b, Supporting Information). When implant size increases while maintaining the same material properties, the results remain consistent, showing no significant impact on bladder deformation. This suggests that both structural design and material softness contribute to the unique functional properties of the scaffold. However, for web-like scaffolds, an increase in size leads to significantly higher mechanical constraints on bladder elastic strain. Both 8-island and 12-island web-like scaffolds (Figure S3a, Supporting Information) exhibit significant differences compared to the control group (Figure S3b,c, Supporting Information). These results highlight that while scaffold size has minimal impact on cross-shaped designs, web-like configurations impose greater mechanical restrictions as they scale up, making them less favorable for bladder integration. Based on the comparative analysis of mechanical performance and scalability, Design 1 (i.e., cross-shaped scaffold) demonstrates the lowest mechanical constraint and highest conformity with bladder expansion. Its minimal impact on bladder deformation across varying sizes supports its suitability for long-term applications. Therefore, Design 1 is selected for further investigation of positional stability under extended cyclic bladder expansion in the following section.

### 2.3. Long-Term Stability of Bladder-integrated Implants

One of the unique biomechanical characteristics of the bladder is its continuous filling and voiding cycles, which exerts dynamic mechanical forces on implanted devices. The average human bladder undergoes approximately six voiding cycles per day.^[40]^ long-term positional stability is a critical requirement for bladder-integrated implants to ensure consistent functionality and durability. To evaluate this, we conduct a long-term cyclic expansion test using a programmable syringe pump, which precisely controls the volume of the bladder model to simulate repeated filling and voiding cycles. The cross-shaped scaffold (Design 1) is selected for this study, where only the bridge sites are selectively bonded to the bladder model.

**Figure 4.**
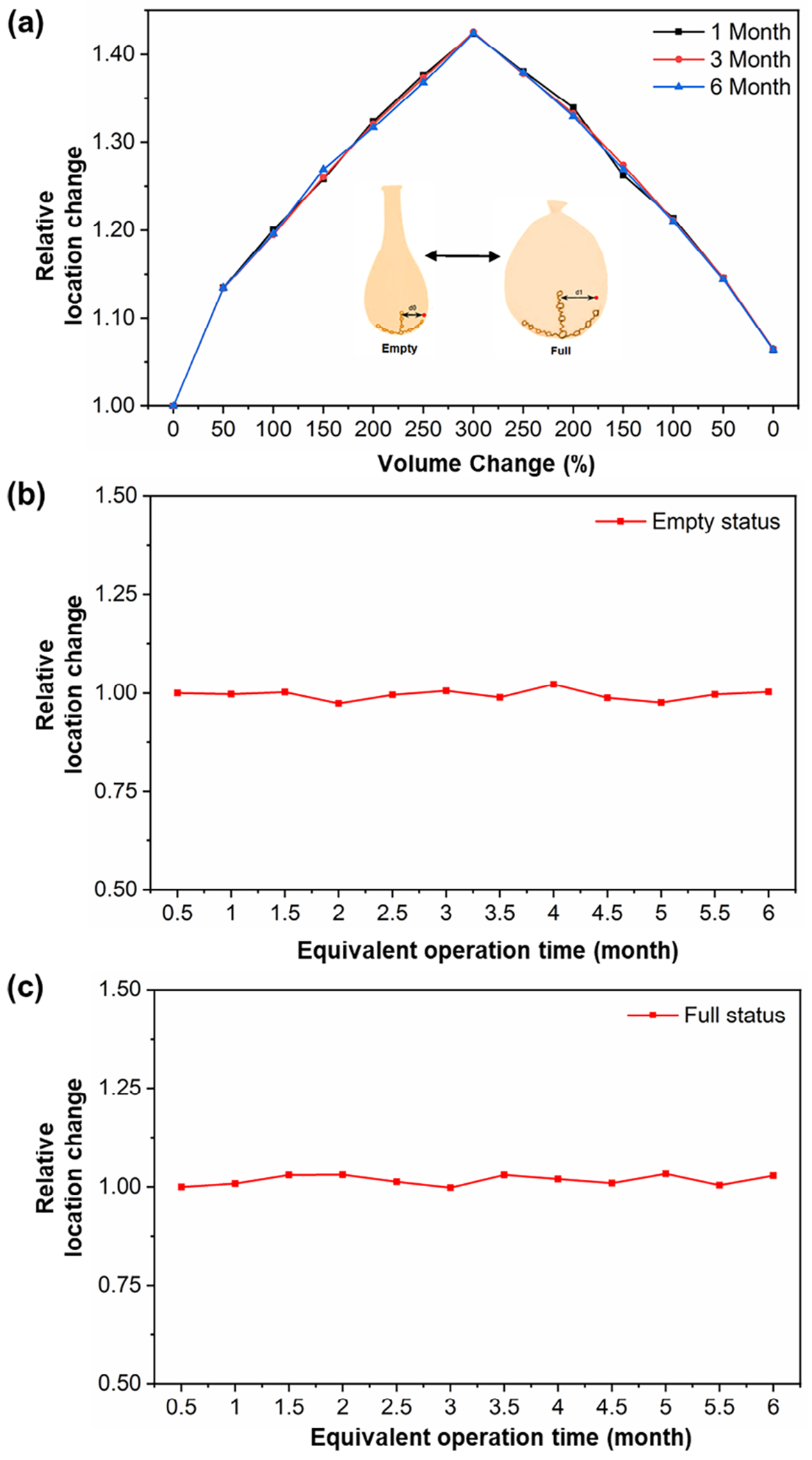
Long-term stability of bladder-integrated implants. a) Loading history of bladder elastic strain during cyclic filling and voiding over 1, 3, and 6 months of simulated bladder activity, showing consistent mechanical response. b) Relative location change of the implant in the empty bladder state after six months of operation (1080 cyclic loading cycles). c) Relative location change of the implant in the full bladder state at 300% volume expansion.

To replicate this in a controlled bench test, we perform 1080 syringe pump cycles that simulate six months of bladder activity. During this period, the scaffolds are tested on a water balloon model undergoing cyclic expansion and contraction with 300% volume change per cycle. The loading history of bladder elastic strain (Figure 4a) remains nearly identical across different time points, with no significant variations observed between 1 month (180 cycles), 3 months (540 cycles), and 6 months (1080 cycles). These results demonstrate the durability and mechanical integrity of the bladder implant over extended use.

To quantitatively assess implant positional stability, we mark a reference point and measure the relative distance between the reference and the implant throughout the experiment. This measurement was used to evaluate implant displacement over time. The relative location change of the implant in an empty bladder state after 1080 cyclic loading cycles (corresponding to six months of simulated operation) is shown in Figure 4b. The results indicate that the average displacement remained approximately constant at 1 mm, with minimal variation over time, confirming consistent implant positioning. Additionally, we analyzed implant displacement when the bladder was fully expanded to 300% of its initial volume (Figure 4c). The results showed similar fluctuations to the empty state, with relative displacement changes ranging between (1.03 mm) and (1.00 mm). These findings further support the conclusion that the implant remains securely positioned and stable under both empty and full bladder conditions throughout the long-term test.

### 2.4. Biocompatibility of Bladder Implants

**Figure 5.**
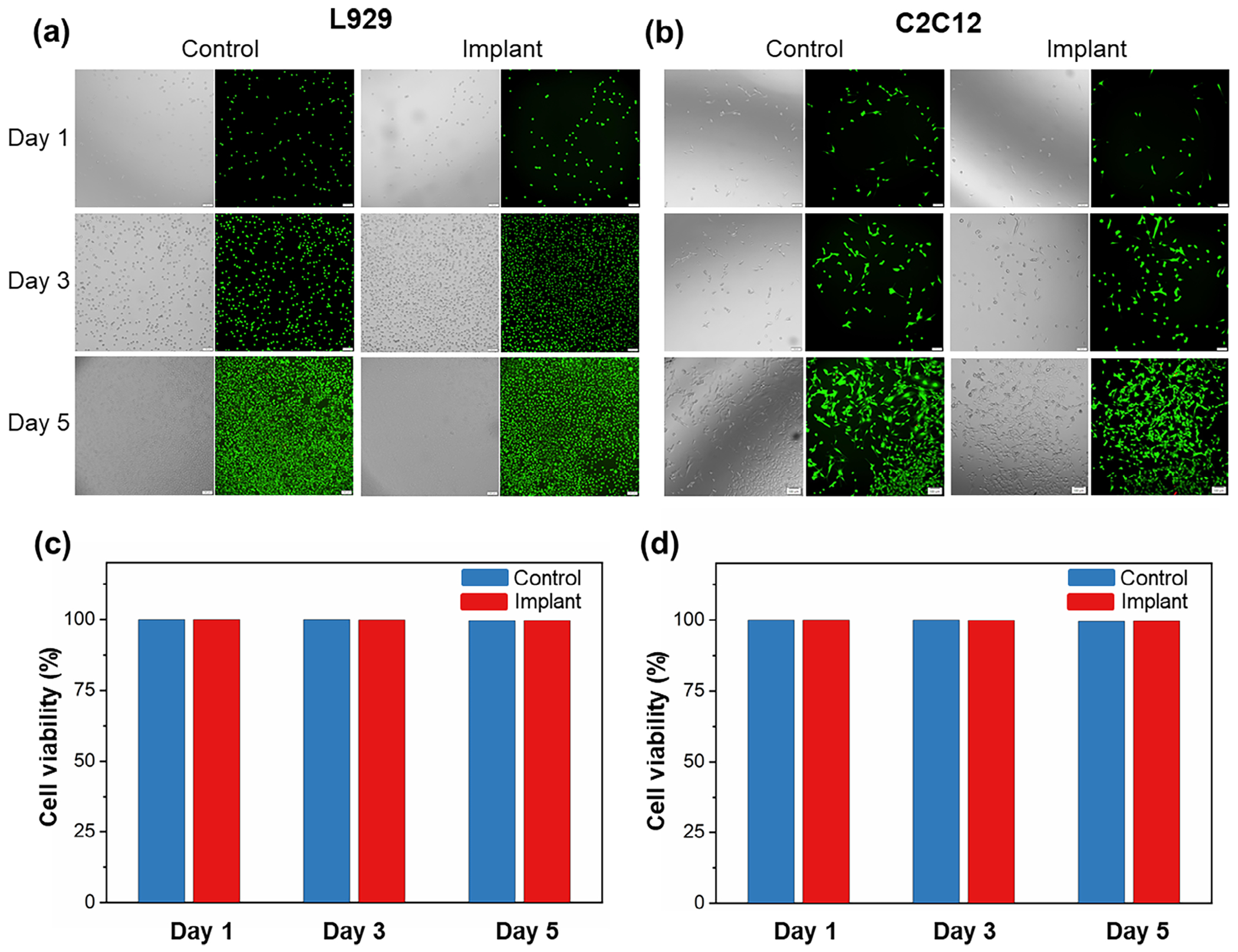
Biocompatibility evaluation of Ecoflex bladder implants. Phase contrast and fluorescence microscopy images of a) L929 fibroblast cells and b) C2C12 myoblast cells cultured in growth medium with Ecoflex implants and without implants (control) at days 1, 3, and 5. Quantitative analysis of c) L929 cell and d) C2C12 cell viability at days 1, 3, and 5.

Biocompatibility is a critical requirement for bladder implants, particularly for in vivo applications, where long-term interaction with biological tissues is necessary. In this study, Ecoflex silicone is used as the scaffold material, and its biocompatibility is assessed through cell viability analysis. To evaluate the cytotoxicity of Ecoflex, L929 mouse fibroblast cells and C2C12 mouse myoblast cells are cultured in growth medium with submerged implants, allowing assessment of potential cytotoxic effects. Cell viability is measured and compared to control groups at days 1, 3, and 5. A live/dead cell staining assay is performed using Calcein-AM and BOBO-3 iodide dual fluorescent stains, followed by fluorescence microscopy analysis. Calcein-AM, a cell-permeant dye, stains live cells green, while BOBO-3 iodide, a cell-impermeant dye, marks cells with compromised membranes in red fluorescence. As shown in Figure 5a,b, both L929 and C2C12 cells cultured in the presence of Ecoflex exhibited pre-dominantly green fluorescence, indicating that cell membranes remained intact, with no signs of toxicity. The fluorescence images further show an increase in cell density over time, suggesting normal cell proliferation and viability throughout the culture period. Although the number of cells reaches confluence by day 5, only a very small fraction of dead cells (red) is visible, demonstrating the non-cytotoxic nature of Ecoflex implants.

To quantitatively assess cell viability, the percentage of viable cells is calculated and presented in Figure 5c,d. The results show that for L929 cells, viability was 100% on day 1, 99.84% on day 3, and 99.57% on day 5. Similarly, for C2C12 cells, viability remains high at 100% on day 1, 99.86% on day 3, and 99.58% on day 5. No statistically significant differences are observed between the Ecoflex-exposed groups and control groups at any time point. Furthermore, cell viability remained consistently above 99% across all conditions, showing that Ecoflex silicone material does not negatively impact cell survival or proliferation. These results show that Ecoflex silicone is a highly biocompatible material suitable for potential in vivo applications, making it an excellent candidate for advanced bladder-integrated bioelectronic implants.

## 3. Conclusion

This work demonstrates the optimal design, mechanical evaluation, and biocompatibility of highly stretchable, bladder-integrated scaffolds for potential use in bioelectronic implants. Among the designs evaluated, cross-shaped scaffolds exhibit the lowest mechanical constraint (≈1.5%) during 300% bladder volume expansion, closely matching the natural deformation of the bladder and ensuring mechanical compatibility. In contrast, web-like and fully integrated configurations show significantly higher constraints (up to 22%), underscoring the importance of geometry in scaffold design. Long-term mechanical stability tests confirm that the scaffolds maintain positional integrity over six months of simulated bladder activity, highlighting their durability for long-term chronic implantation. Furthermore, cytocompatibility assays using L929 fibroblast and C2C12 myoblast cells demonstrate that Ecoflex-based implants exhibit very high biocompatibility (>99.5% cell viability over five days), with no significant cytotoxic effects.

Looking ahead, future studies will extend this platform toward physiological validation using ex vivo and in vivo bladder models to better assess tissue-device interactions under realistic voiding conditions. The development of advanced mounting strategies, such as bioadhesive interfaces, will further support stable and biocompatible integration without relying on sutures. Evaluations under cyclic mechanical strain, along with strategies to mitigate foreign body response and fibrotic encapsulation, will be critical for ensuring long-term functionality. Overall, this study lays a solid foundation for the integration of microelectronics with soft, adaptive scaffolds, enabling the development of next-generation bladder implants for continuous health monitoring and neuromodulation therapy.

## 4. Experimental Section

### Materials:

The designs were 3D printed using Forms 3+ 3D printer from Formlabs (Massachusetts, USA). The resins used in the 3D printer for fabricating the negative molds are formlabs photopolymer resin black V4 from Formlabs company (Massachusetts, USA). The elastomers consisted of Ecoflex 00–20 FAST from SMOOTH-ON company (Pennsylvania, USA) and Polydimethylsiloxane (PDMS) Sylgard 184 from Dow Inc (Michigan, USA). The molds were filled with elastomers by 1 cc/ml syringes with 27Gx1/2″ needle from Air-Tite Co., Inc. (Illinois, USA). The water balloons that mimicked the structure of the rat bladder were purchased from a local supermarket. The balloons connected to the NE-1000 1-channel Syringe Pump from New Era Pump Systems (New York, USA) for numerous fillings and voiding of the balloons.

### Design::

The 2D designs were created using AutoCAD 2024 software. The 3D structures were extruded from 2D designs using the same software. The resulting 3D platform was exported to .stl file and opened with PreForm software to further edit and assign precision of the printing layers.

### Scaffold Fabrication:

The negative molds were designed with AutoCAD software. The width of traces was 0.6 mm with serpentine amplitude of 1.3 mm and wave period of 2.6 mm. The dimensions from one end electrode to the other was 24 mm for 8 and 36 mm for 12 island structures, and 48 mm for 16 island design. The molds were then 3D printed with SLA 3D printer Form 3+ from Formlabs company. The printed platforms were rinsed in IPA for 30 min and cured under UV light for 45 min. The Ecoflex 00–20 FAST and PDMS Sylgard 184 were used for scaffolds. The molds’ traces were then sprayed with Ease Release 200 to lower the adhesion of its surface. The Ecoflex agents were mixed with 1:1 mixture ratio. When mixed, the elastomer was carefully poured in the traces of the negative mold using a syringe with a 27-gauge flat-head needle. The molds containing the uncured Ecoflex were put in vacuum to dispose of the potential bubbles trapped inside the traces that could interfere with the integrity and uniformity of the scaffolds. After 30 min to 1 h of curing in room temperature, the scaffolds were extracted using a sharp-headed tweezer. As for the PDMS scaffolds, the PDMS was used with part A and part B mixed at the weight ratio of 10:1. The same process of Ecoflex was conducted for the PDMS and it was left to cure and solidify overnight. The scaffolds’ geometries were measured using optical microscopy (Olympus BX53 M).

### Mechanical Characterization:

The scaffolds were carefully placed on the dome of the water balloons that would mimic the structure and stretch-ability of rat bladder. Two points on the balloons were marked with a marker to measure the distances between the two when the balloon was empty versus filled. Then the balloons that were connected to a syringe pump (1000-US / SyringeONE, USA) were filled with 15 ml of water at the speed of 1 mL min^−1^. Photographs were taken at intervals corresponding to changes in balloon volume, ranging from 0% to 300% in increments of 50%. Each design was repeated three times. To simulate normal bladder activity over six months, using software Pumping Program Generator and set 1080 pumping cycles for long-term functionality testing.

### Live/Dead Cell Staining Assay:

The cytocompatibility of Ecoflex was evaluated using a live/dead cell staining assay with mouse fibroblast cells (L929) and murine myoblasts (C2C12), both obtained from the American Type Culture Collection (ATCC). C2C12 cells were cultured in a growth medium consisting of Dulbecco’s Modified Eagle Medium (DMEM, Corning) supplemented with 10% Fetal Bovine Serum (FBS, Sigma–Aldrich) and 1% Penicillin-Streptomycin (Sigma–Aldrich), while L929 cells were maintained in Eagle’s Minimum Essential Medium (EMEM, Corning) with the same supplements. Ecoflex samples (5 mm in diameter) were sterilized with 70% ethanol and rinsed with deionized water. C2C12 and L929 cells were seeded in 24-well plates at densities of 3000 and 20 000 cells per well, respectively, and co-cultured with Ecoflex in an incubator at 37 °C and 5% CO_2_. Cells cultured without Ecoflex served as control groups. Cell viability was assessed on days 1, 3, and 5 using the live/dead cell imaging kit (R37601, Thermo Fisher) following the manufacturer’s protocol. Fluorescence microscopy (IX83, Olympus) with FITC and TXRED filters was used to visualize the samples. Cell viability was quantified using ImageJ software by analyzing live (green fluorescence) and dead (red fluorescence) cells.

### Statistical Analysis:

The comparison of means between different groups of numerical variables was performed using a one-way ANOVA. *P* value less than 0.05 was considered statistically significant. Statistical analyses were performed using Stata 17 (StataCorp LLC). Significance level: *p < 0.05, **p < 0.01, ***p < 0.001.

## Supplementary Material

Supplementary Information

Supporting Information is available from the Wiley Online Library or from the author.

## Data Availability

The data that support the findings of this study are available from the corresponding author upon reasonable request.
