# Supplementary material for: Design of Soft, Stretchable Bladder-Integrated Scaffolds for Advanced Bioelectronic Implants
Source: Adv Mater Technol. Author manuscript; Available in PMC 2026 Apr 28. (PMC13120774; doi:10.1002/admt.202500552)
Supplement: Supplementary Information [file NIHMS2158894-supplement-Supplementary_Information.docx]

**Supporting Information**

**Design of Soft, Stretchable Bladder-Integrated Scaffolds for Advanced Bioelectronic Implants**

Yifan Wang^1†^, Ali Garmroudi^1†^, Chang Liu^1^, Philippe Zimmern^2^, Zhengwei Li^1,3,4*^

^1^Department of Biomedical Engineering, University of Houston, Houston, Texas

^2^Department of Urology, The University of Texas Southwestern, Dallas, Texas

^3^Department of Biomedical Sciences, The Tilman J. Fertitta Family College of Medicine, University of Houston, Houston, Texas

^4^Institute of Muscle Biology and Cachexia, University of Houston, Houston, Texas

†Yifan Wang and Ali Garmroudi contributed equally to this work.

*Corresponding author: [zli65@central.uh.edu](mailto:zli65@central.uh.edu)

Table S1: Summary of Structural Designs, Materials, and Stretchability

| **Structural Design** | **Materials Used** | **Stretchability (% strain)** | **Application Domain** | **Reference** |
| --- | --- | --- | --- | --- |
| Serpentine | PDMS + Au/PI | 32% | Skin, brain, heart, muscle | [35] |
| porous | Ag NW + porous SEBS | 100% | Skin | [36] |
| Wavy | CNT/PDMS | 100% | Wearable electronics | [37] |
| Origami pattern | Ag NW+TPU | 400% | High areal coverage | [38] |
| Kirigami mesh | Ag ink | >150% | Skin wearable electronics | [39] |
| Soft mesh bladder-integrated scaffold (this work) | Ecoflex, PDMS | ~50% planar (300% volume) | Bladder-integrated electronics | This work |


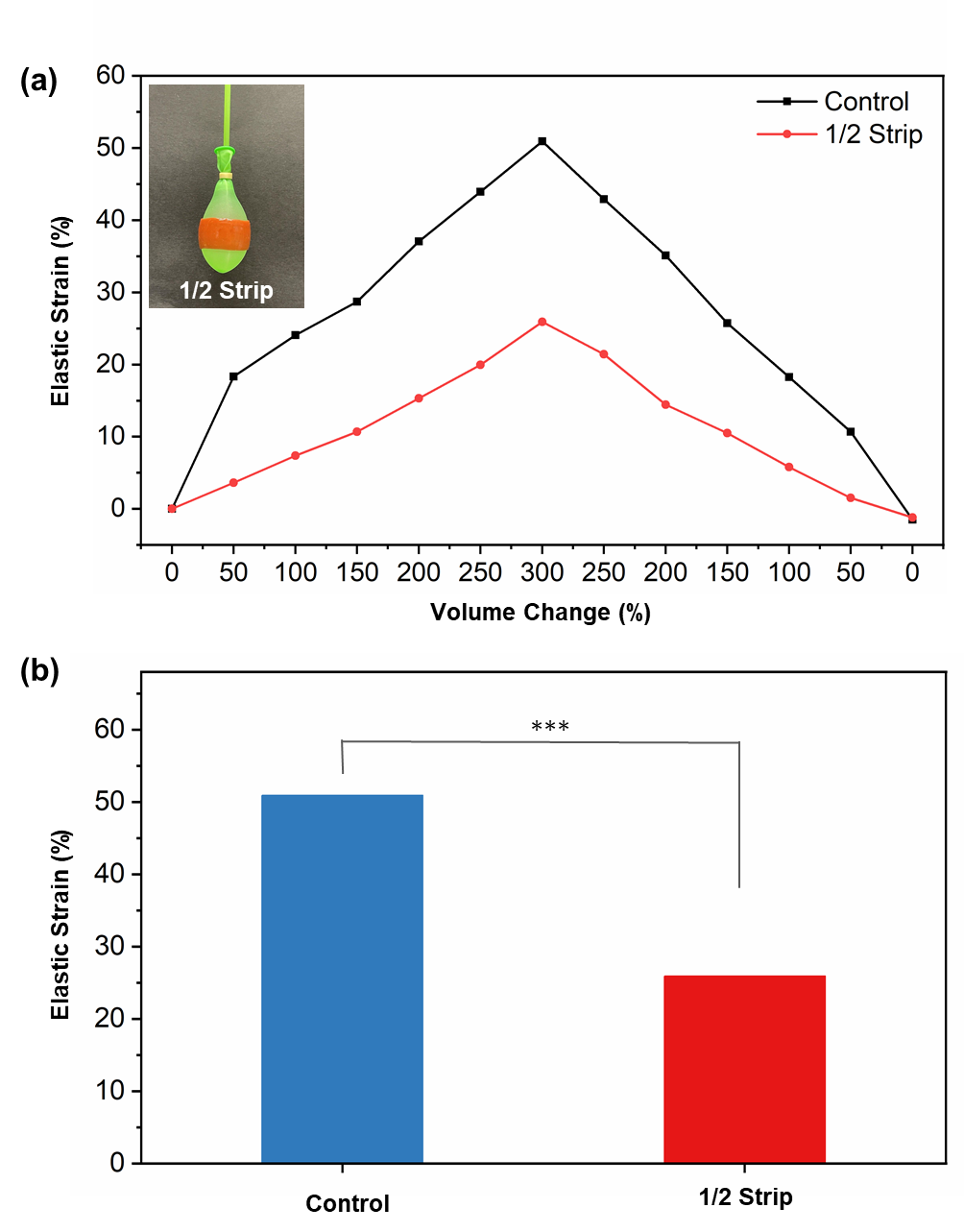


**Figure S1. Impact of strip-shaped scaffold on bladder expansion. (a)** Loading history of bladder elastic strain for the control (no implant) and the strip-shaped scaffold (insert) which has a width of 17mm, wrapping around the center of the bladder model. **(b)** Comparison of elastic strain between the control (no implant) and the strip-shaped scaffold.


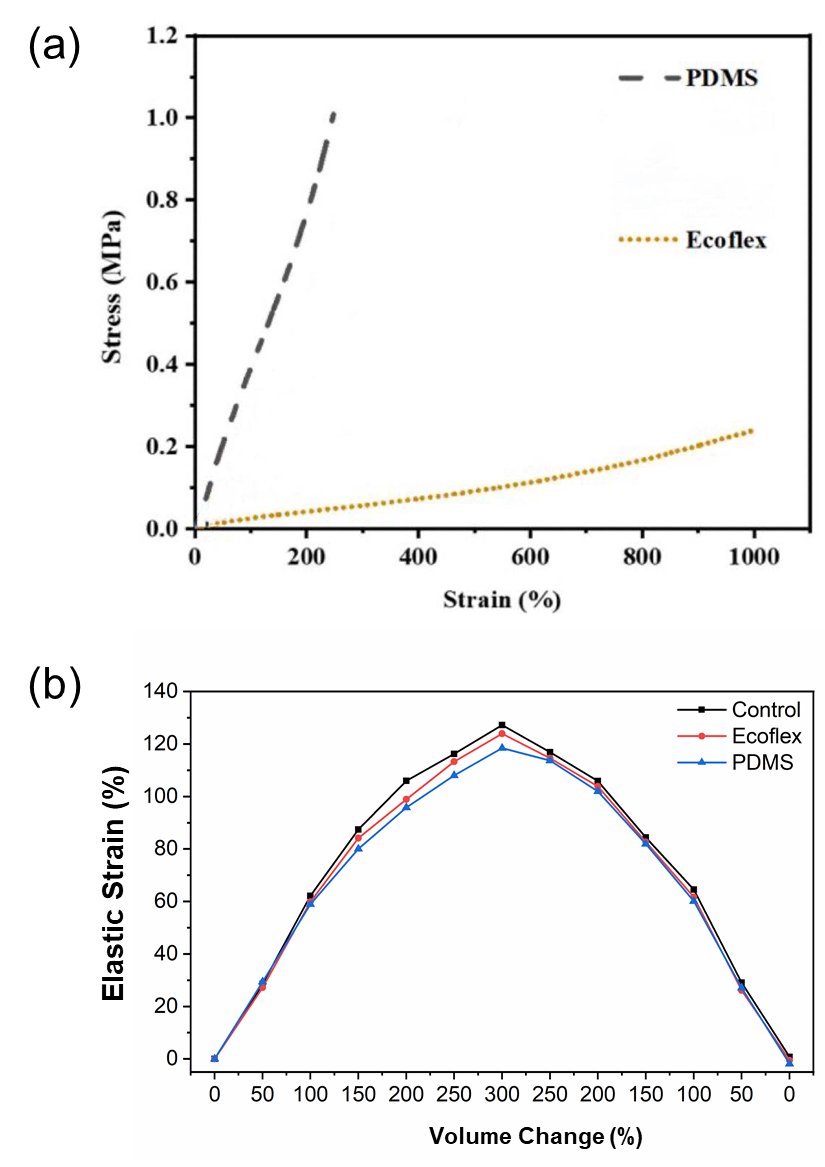


**Figure S2. Effect of material stiffness on bladder expansion.** (a) Representative stress–strain curves of two commonly used silicone-based elastomers, Sylgard 184 PDMS and Ecoflex. Data adapted from Ref [1], showing the significant difference in Young’s modulus between the two materials. PDMS (10A:1B) exhibits a higher modulus (~1.56 MPa), whereas Ecoflex (1A:1B) shows a much lower modulus (~46 kPa), closely aligning with the compliance of soft biological tissues such as the bladder (b) Bladder elastic strain during expansion and contraction for scaffolds made of PDMS and Ecoflex, compared to the control (no implant).


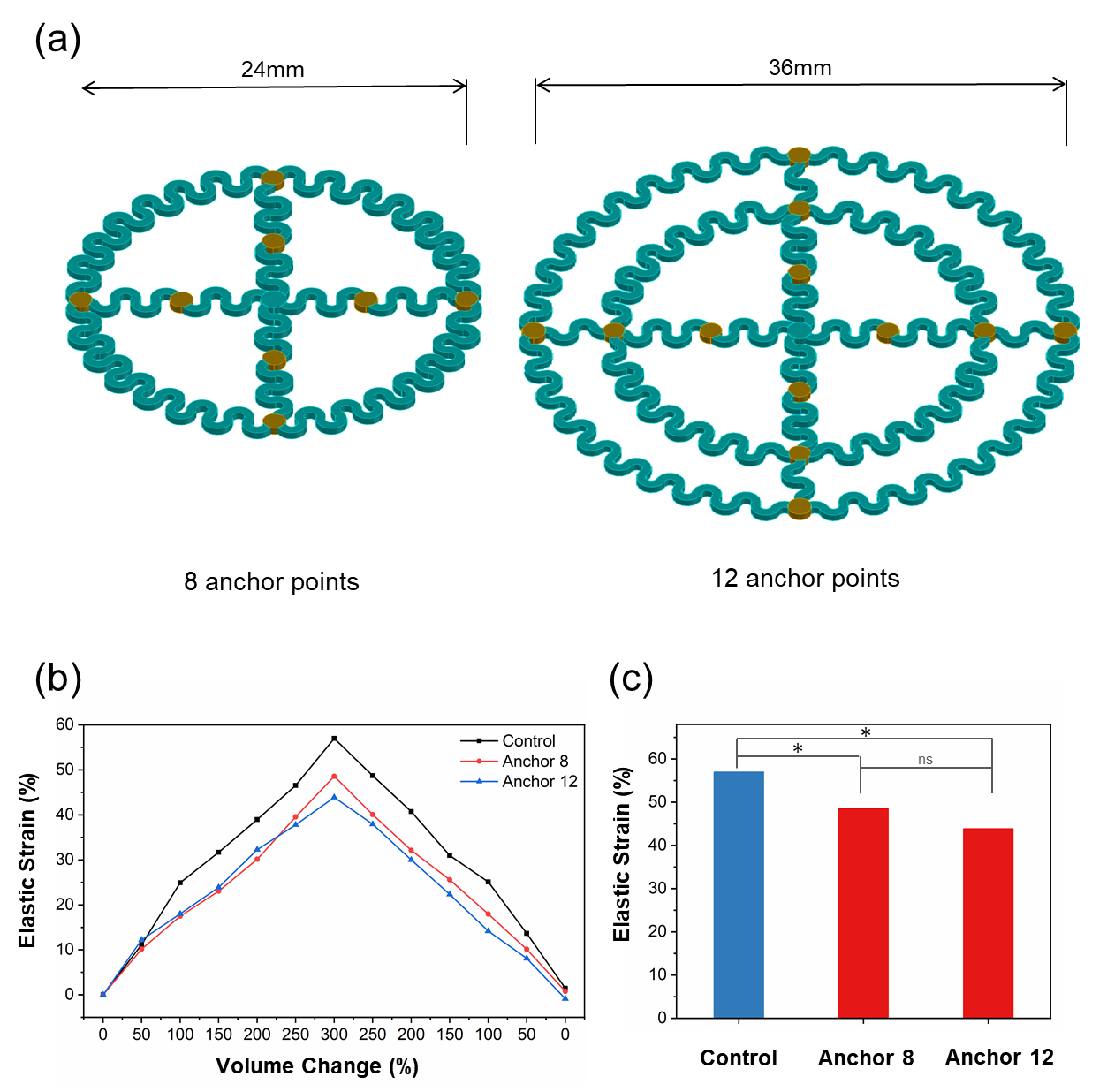


**Figure S3. Impact of web-like scaffold size on bladder expansion.** (a) Schematic of web-like scaffold designs with different sizes, featuring 8 islands (Anchor 8) and 12 islands (Anchor 12) as anchor points. (b) Loading history of bladder elastic strain for different scaffold sizes and the control (no implant) during the expansion and contraction process. (c) Comparison of elastic strain at 300% volume expansion across different scaffold sizes, showing increased mechanical constraints in larger web-like designs.

**Reference**

[1]A. N. Kouediatouka, Q. Liu, F. J. Mawignon, W. Wang, J. Wang, C. Ruan, K. F. H. Yeo, G. Dong, *Applied Surface Science* **2023**, *635*, 157675.
